# Deconvolution of Single-Organism Omics Resolves Cellular and Tissue Dynamics during Aging

**DOI:** 10.17912/micropub.biology.001989

**Published:** 2026-04-13

**Authors:** Youngjun Park, Adam Antebi

**Affiliations:** 1 Department of Molecular Genetics of Ageing, Max Planck Institute for Biology of Ageing, Cologne, North Rhine-Westphalia, Germany; 2 Cologne Excellence Cluster on Aging and Aging-Associated Diseases, Cologne, North Rhine-Westphalia, Germany

## Abstract

The deconvolution of bulk omics data provides enhanced resolution of cell populations within samples and has been widely adopted, particularly in cancer biology. In this study, we applied deconvolution approaches to both transcriptomic and proteomic datasets at single-organism resolution in *C. elegans* , enabling us to infer cellular and tissue contributions to the whole transcriptome or proteome. Our results demonstrate that deconvolution-derived cellular and tissue proportions can serve as robust proxy readouts for dynamic changes in cells and tissues during aging.

**Figure 1. Deconvolution of single-worm omics data:** **A** . Schematic overview of the single-worm transcriptomic (swRNAseq) and proteomic (swProteomics) workflows, followed by cellular deconvolution using a marker-gene reference dataset obtained from single-cell RNA sequencing of L4 worms.&nbsp; **B, D, F** . Predicted cellular and tissue proportions shown as stacked bar plots. **B** . Deconvolution results from swRNAseq of the *C. elegans* N2 strain. Samples from Days 1–12 were aggregated, and mean proportions are presented. **D** . Predicted cellular and tissue proportions from swRNAseq of the *glp-1* mutant strain. **F** . Predicted cellular and tissue proportions derived from swProteomics of the N2 strain. **C, E, G** . Violin plots showing correlation analyses between biological replicates collected at the same day. Between-day correlations among randomly paired samples are indicated by a red line. **C** . Correlation distribution for swRNAseq of the N2 strain. **E** . Correlation distribution for swRNAseq of the *glp-1* strain. **G** . Correlation distribution for swProteomics profiles of the N2 strain. **H** . *C. elegans* cellular and tissue proportions measured by nuclei count and total estimated cell volume. **I.** Correlation with ground truth for the number of *C. elegans* cells (nuclei fraction) and cell volume measurements. The correlation between total cell volume and nuclei count per tissue is indicated by a red line. **J.** Example of outliers in the comparison between ground truth and predictions.

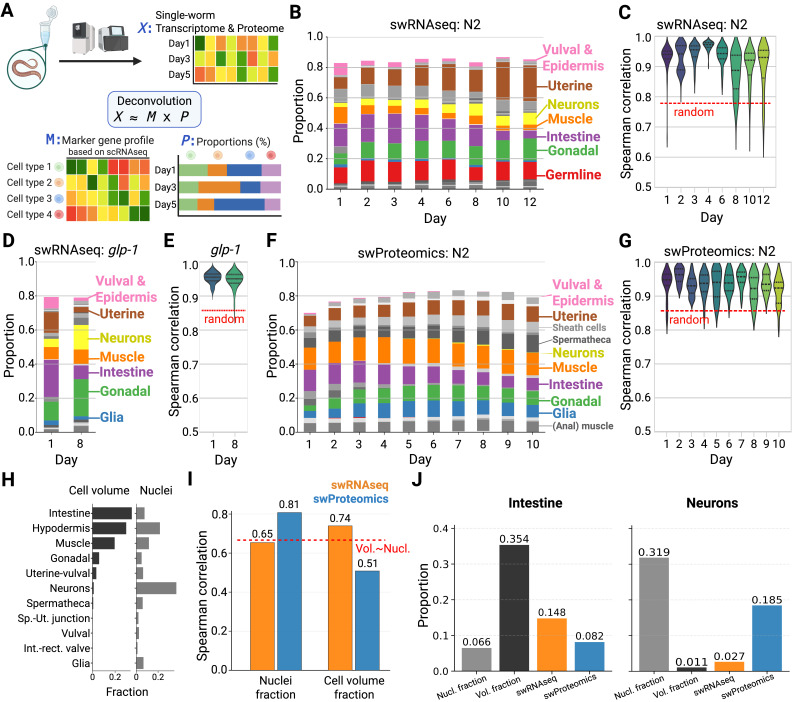



## Description

Deconvolution has been explored in bulk RNA sequencing analyses, particularly in cancer biology, where it is widely used to characterize immune infiltration within tumors (Newman et al., 2019). This analytical framework uses linear models to infer cell proportions based on the expression profiles of known cell types. It enhances the value of bulk RNA sequencing data by providing an additional perspective for interpreting samples.

Single-worm RNA sequencing (swRNAseq)&nbsp;and single-worm proteomics (swProteomics) offer powerful approaches for capturing molecular variation at the level of individual organisms. By measuring omics from single worms, these methods enable researchers to assess inter-individual differences and characterize organism-specific responses to genetic or environmental perturbations (Eder et al., 2024; Zhu et al., 2024). As a result, single-worm omics technologies have become valuable tools in genetics and systems biology, providing a high-resolution view of how genotype, age, and various perturbations shape molecular phenotypes in *C. elegans* .

To exploit this single-organismal data, which is fundamentally a bulk-cell omics measurement, we applied a deconvolution method (See Methods) to the swRNAseq data. This allowed us to investigate individual cell- or tissue-level dynamics during aging, utilizing publicly available data ( Figure 1A ).&nbsp;The deconvoluted swRNAseq data from *ad libitum* aging reveal the relative shrinkage of several somatic tissues: intestine (decreasing from Day 3: 19.3% to Day 12: 5.2%), muscle (Day 1: 10.7% to Day 12: 3.6%), and vulval/epidermis (Day 1: 7.9% to Day 12: 1.2%). Concurrently, the relative transcriptional contribution of (somatic) gonadal (Day 1: 7.2% to Day 12: 14.4%), neurons (Day 1: 2.7% to Day 12: 7.8%), and uterine (Day 1: 7.5% to Day 12: 23.3%) expand proportionally ( Figure 1B ). The proportions of all the other cell types are shared with the analysis code (See Extended Data).

Previously, various studies of aging in *C. elegans* have revealed that the intestine undergoes pronounced age-related deformation (Herndon et al., 2002; Wang et al., 2022), which is consistent with the deconvolution result. Progressive intestinal pathologies have been reported (McGee et al. 2011), including loss of nuclei, degradation of microvilli, and substantial changes in intestinal size, shape, and cytoplasmic content. During the aging process, *C. elegans* exhibits progressive loss of myofibril integrity, loss of cytoplasmic muscle content, and a progressive decline in locomotor ability. Neurons are post-mitotic and remarkably resilient compared to other somatic tissues, which may explain the relative increase in transcriptional contribution of neurons, when the intestine and muscle deform and decline during aging.&nbsp;The increased proportion of (somatic) gonadal and uterine tissue may reflect a disease syndrome that drives multimorbidity and contributes to late-life mortality (Ezcurra et al., 2018). Additionally, the heightened gonadal signal in aging worms may correlate with egg retention (bagging), uterine tumors, or an accumulation of unlaid oocytes that continue to transcribe germline/uterine genes, thereby overwhelming the transcriptional signal of the dying somatic tissues (muscle/epidermis) in the sequencing data (Zimmerman et al., 2015). Conversely, the shrinkage of the vulval/epidermis can be explained by age-associated vulval integrity defects, which encompass the physical deterioration and transcriptional decline of the hypodermal layer (Leiser et al., 2016).

To assess the robustness of our deconvolution method, we calculated pairwise correlations among samples within the same time points. The average Spearman correlation coefficient exceeded 0.9, demonstrating that the deconvolution approach yields highly consistent and reproducible proportion estimates, notably higher than the average correlation of 0.778 observed across 100,000 randomly paired samples across different days ( Figure 1C ). The same results were observed with Pearson correlation as well (intra-day: 0.99 and random: 0.80; See Extended Data).

We were interested to determine whether a strain with depleted germline cells might affect the robustness of the model and exhibit aging phenotypes similar to those observed in wild types, so we examined the *glp-1* strain. In the *glp-1* mutants, we observed a shrinking intestine (Day 1: 21.7% to Day 8: 8.0%) accompanied by an expansion of (somatic) gonadal cells (Day 1: 11.0% to Day 8: 21.8%). Neuronal proportions also increased (4.8% to 14.4%), whereas vulval and epidermal cell proportions decreased (Day 1: 6.7% to Day 8: 1.9%) ( Figure 1D ). The average Spearman correlation between paired samples at the same time points was 0.94, further demonstrating the robustness and reproducibility of the deconvolution approach ( Figure 1E ).

Although deconvolution methods were originally developed for RNA sequencing datasets, recent studies have shown that they can also be successfully applied to proteomic data. However, in the context of poteomics, comprehensive cell type–specific proteomic markers for *C. elegans* are not yet available. Consequently, the only practical strategy at present is to rely on marker genes identified from single-nucleus RNA sequencing studies.

In this work, we explored the feasibility of applying such deconvolution approaches to swProteomics datasets. Recent advances in swProteomics now enable proteome analysis at true individual-worm resolution, making it possible to investigate dynamic changes at the proteomic level. The proteomics results reveal tissue proportion dynamics similar to those observed in our swRNAseq data. Specifically, we find a decrease in intestinal cell proportions with age (Day 1: 12.5% to Day 10: 7.2%) similar to the result from transcriptomics ( Figure 1F ). Gonadal and uterine proportions showed an expansion (Day 1: 3.2% to Day 10: 8.0%, Day 1: 6.2% to Day 10: 9.1%), although the increase was less pronounced than that observed in the single-worm RNA sequencing results. The average correlation between paired samples at the same time points was 0.935, compared to 0.857 for randomly paired samples across different time points, highlighting the consistency and reliability of the deconvolution results ( Figure 1G ).&nbsp;Some cell types, such as neurons, are not represented here due to the limited proteome coverage of the available single-worm dataset (~3,500 proteins detected in Zhu et al.), as well as the applied marker gene thresholds. We expect that deconvolution will provide more accurate tissue proportions when higher-depth proteomics samples become available.

We obtained the number of nuclei and estimated cell volumes for young adult *C. elegans* from the work by Froehlich et al. (2021; Figure 1H ). To test aspects of our model, correlation analysis was performed to compare them with the deconvolution predictions. The analysis suggests that the deconvolution results are moderately correlated with the ground truth of cell volume and number of nuclei, although this omics-based proportion does not perfectly match either nuclei counts or cell volumes ( Figure 1I ). Notably, cell-type–specific differences are present in the results. This becomes apparent when examining outlier tissues such as neurons and intestine, which show greater discrepancies ( Figure 1J ). Neurons account for nearly 30% of the total cells in *C. elegans* ; however, both protein- and mRNA-based deconvolution estimate only marginal proportions. This discrepancy may result from the limited sensitivity of mRNA and protein measurements. In contrast, the intestine shows the opposite pattern: the cell volume fraction is greater than the nuclei number fraction, while the deconvolution results appear to reflect nuclei fractions more closely.

Despite current limitations, our proof-of-concept analysis demonstrates that protein-level deconvolution using transcriptome-derived marker genes is both feasible and informative. As proteomics technologies become increasingly accessible and scalable, this approach will offer novel insights into the cellular and tissue composition of individual organisms. It is important to note that although our deconvolution results are supported by known pathological changes observed during worm aging, this approach does not directly reflect the absolute number of cells in an individual worm, as discrepancies may exist between ground-truth cell numbers and the detectable amounts of mRNA and proteins across different cell types. Therefore, these measurements should primarily be used to trace and compare trends, such as aging trajectories, rather than being interpreted as absolute quantitative values. To understand the underlying biological mechanisms behind the observed trends, follow-up experiments will be required.

We expect that extending the capabilities of swProteomics, which has strong potential for high-throughput scalability, will enable researchers to characterize biological variation with much greater resolution, uncover subtle physiological differences, and ultimately support more detailed mechanistic studies.

## Methods

**Deconvolution model**

A wide range of deconvolution methods have been developed to date, and any of these approaches can, in principle, be applied in this analysis. In this work, we implemented a simple linear model using linear support vector regression (SVR), which is conceptually similar to the framework employed by CIBERSORTx (Newman et al., 2019). The algorithm takes as input the total bulk expression profile along with the expression signatures of marker genes, and estimates the proportion of each cell type required to reconstruct the observed bulk expression. The SVR model is primarily optimized through two parameters, C and ε, which control the regularization strength and the width of the epsilon-insensitive tube, respectively. Through empirical testing, we identified optimal performance at C = 0.001 and ε = 0.01, within the parameter ranges of C = 0.0001–0.1 and ε = 0.001–0.5.

Marker gene information was derived from single-nucleus RNA sequencing data obtained from L4-stage *C. elegans* &nbsp;(Ghaddar et al., 2023). From this dataset, the top 100 marker genes for each cell type were selected and used to infer cell-type proportions in both single-worm RNA sequencing and single-worm proteomics datasets. Due to the lower sensitivity and technical limitations of proteomics, relatively few proteins are detected, and many transcript-derived marker genes are not represented at the protein level. Therefore, the marker-gene list was filtered prior to deconvolution. For single-worm proteomics, we applied thresholds to filter cell types for estimation, requiring a minimum of five available marker genes and a minimum marker score of 0.08 per marker gene. For single-worm RNA sequencing, we used a minimum marker gene count of 0 and a minimum marker score of 0.08 per marker gene.

For ground-truth comparison, cell nuclei counts and volume data were obtained from young adult *C. elegans* (Froehlich et al., 2021). Thirteen major cell types and tissues were curated (intestine, hypodermal cells, muscle, gonadal, uterine-vulval, neurons, spermatheca, sp-ut junction, vulval, int-rect valve, glia, distal tip, and coelomocytes). A correlation analysis was performed on the results using a minimum marker gene count of 0 and a minimum marker score of 0.08 per gene.

**Data availability**

The single-worm RNA sequencing data set was obtained with the BioProjectID: PRJNA1015633 from NCBI Sequence Read Archive (Eder et al., 2024). The single-worm proteomics dataset was obtained with PXD039330 from ProteomeXchange Consortium via the PRIDE (Zhu et al., 2024).&nbsp;The ground-truth cell nuclei counts and volume data for young adult *C. elegans* were obtained from the supplementary data of Froehlich et al. (2021).

## Data Availability

Description: Python analysis code for the deconvolution of single-worm transcriptomic and proteomic data, including intermediate results and figures generated using different parameter configurations.. Resource Type: Software. DOI: https://doi.org/10.22002/xhma5-zsq45
